# A highly conserved mechanism for the detoxification and assimilation of the toxic phytoproduct L-azetidine-2-carboxylic acid in *Aspergillus nidulans*

**DOI:** 10.1038/s41598-021-86622-3

**Published:** 2021-04-01

**Authors:** Ada Biratsi, Alexandros Athanasopoulos, Vassili N. Kouvelis, Christos Gournas, Vicky Sophianopoulou

**Affiliations:** 1 Microbial Molecular Genetics Laboratory, Institute of Biosciences and Applications, National Centre for Scientific Research, Demokritos (NCSRD), Athens, Greece; 2 Department of Genetics and Biotechnology, Faculty of Biology, National and Kapodistrian University of Athens, Athens, Greece; 3 Light Microscopy Unit, Institute of Biosciences and Applications, National Centre for Scientific Research, Demokritos (NCSRD), Athens, Greece

**Keywords:** Metabolic engineering, Molecular engineering, Cellular imaging, Model fungi, Bioinformatics, Classification and taxonomy, Eukaryote, Gene expression, Gene regulation, Genetic interaction, Microbial genetics, Cellular microbiology, Fungi, Microbial genetics

## Abstract

Plants produce toxic secondary metabolites as defense mechanisms against phytopathogenic microorganisms and predators. L-azetidine-2-carboxylic acid (AZC), a toxic proline analogue produced by members of the Liliaceae and Agavaciae families, is part of such a mechanism. AZC causes a broad range of toxic, inflammatory and degenerative abnormalities in human and animal cells, while it is known that some microorganisms have evolved specialized strategies for AZC resistance. However, the mechanisms underlying these processes are poorly understood. Here, we identify a widespread mechanism for AZC resistance in fungi. We show that the filamentous ascomycete *Aspergillus nidulans* is able to not only resist AZC toxicity but also utilize it as a nitrogen source via GABA catabolism and the action of the AzhA hydrolase, a member of a large superfamily of detoxifying enzymes, the haloacid dehalogenase-like hydrolase (HAD) superfamily. This detoxification process is further assisted by the NgnA acetyltransferase, orthologue of Mpr1 of *Saccharomyces cerevisiae*. We additionally show that heterologous expression of AzhA protein can complement the AZC sensitivity of *S. cerevisiae*. Furthermore, a detailed phylogenetic analysis of AzhA homologues in Fungi, Archaea and Bacteria is provided. Overall, our results unravel a widespread mechanism for AZC resistance among microorganisms, including important human and plant pathogens.

## Introduction

L-Azetidine-2-carboxylic acid (AZC) is a toxic analogue of L-proline (L-Pro) produced by members of the Liliaceae, Agavaceae, Asparagaceae, Fabaceae families and by several *Beta vulgaris* cultivars, such as beetroot or sugar beet^1–3^. Although it is not fully understood why some plants produce toxic compounds like AZC, it has been suggested that such compounds can serve as a defense mechanism, by poisoning predators, pathogens or competitors, thus protecting against infections and consumption, or impeding the growth of neighboring plants^4–6^. AZC defense mechanism relies on the stereochemical similarity of the non-proteinogenic imino acid AZC with the proteinogenic L-Pro, which differ by only one C atom^7^. AZC is incorporated into proteins during protein synthesis, as it can be loaded on L-proline-tRNA instead of L-Pro, a phenomenon that has been characterized as protein misincorporation or amino acid mimicry^8–10^. With one C atom less, AZC is less flexible than L-Pro, resulting in polypeptides with a capability of rotation reduced by 15°^8,11^. These changes eventually affect the folding and the tertiary structure of the proteins in which AZC is incorporated, resulting in non-functional, misfolded proteins. The accumulation of these protein products becomes deleterious and, finally, obstructs cell proliferation and growth^12–14^.

Mechanisms of resistance to AZC have been described in plants, fungi and bacteria^15^. It has been suggested that AZC-producing plants protect themselves from AZC auto-toxicity, by avoiding the loading of AZC on L-Pro-tRNA via at least three mechanisms: (a) their L-Pro-tRNA activating enzymes are highly specific and discriminate between L-pro and AZC (b) AZC is subcellularly excluded from protein-synthesis sites^16^ and (c) some plants possess an efficient tRNA synthetase editing system, able to remove AZC loaded on L-Pro-tRNA^9^.

In fungi, it has long been shown that some strains of the budding yeast *Saccharomyces cerevisiae* with increased intracellular levels of L-Pro are resistant to AZC^17^. Moreover, a specific strain, Σ1278b, was shown to detoxify AZC through the function of Mpr1/2 acetyltransferases^18^. Mpr1/2, members of the *N*-acetyl-transferase superfamily, were shown to catalyze the acetylation of AZC to produce *N*-acetyl-AZC, a compound that is no longer recognized by tRNA synthetases and is therefore non-toxic to the cell^19^. However, AZC detoxifying activity of Mpr1/2 has been so far identified only in the Σ1278b strain of *S. cerevisiae* and in very few other yeast species^20,21^.

In bacteria, an additional mechanism of AZC detoxification has been described in a *Pseudomonas* strain, which was isolated from soil surrounding the roots of the AZC-producing plant *Convallaria majalis*, a member of the Liliaceae family. This bacterium was found to possess a hydrolase (AC-hydrolase) able to detoxify AZC^22^. The underlying molecular mechanism leads to the periplasmic hydrolysis of AZC to 2-hydroxy-4-aminobutyrate, a substance that could then be transported into the cytoplasm by the action of a GABA transporter homologue, where it subsequently undergoes transamination^22^.

In order for AZC to be toxic, its intracellular accumulation is essential. In yeast, this is known to occur via active transport by three broad specificity amino acid transporters (Agp1, Gnp1 and the general amino acid permease, Gap1) as well as the specialized L-Pro transporter, Put4^23^. In a previous report, we described the specificity of PrnB, the Put4 orthologue in *A. nidulans,* and we demonstrated that, contrary to Put4, PrnB is highly specific to L-Pro and does not recognize AZC. Remarkably, during this work we noticed that *A. nidulans* shows resistance to AZC, for which its Mpr1 orthologue NgnA (formerly recorded as Ngn2^24^) is not necessary^25^.

In the present study, we characterize the mechanism underlying resistance of potentially several fungi to AZC, using *A*. *nidulans* as a model organism. We report that *A. nidulans* possesses the ability not only to detoxify AZC, but also to utilize it as a poor nitrogen source, by its catabolism via the GABA pathway^25–27^. We show that AzhA, an AC-hydrolase orthologue, is necessary and sufficient for AZC detoxification and assimilation. An in silico analysis revealed that AzhA is a member of the haloacid dehalogenase-like hydrolase (HAD) super-family, a large group of enzymes with diverse substrate specificity that has been associated with the detoxification of toxic compounds and xenobiotics^28^. Furthermore, we report that NgnA acetyltransferase is involved in AZC detoxification and we provide a detailed phylogenetic analysis of AzhA amino acid sequence across the three domains of life.

## Results

### AZC detoxification and assimilation in *A. nidulans*

**Figure 1:**
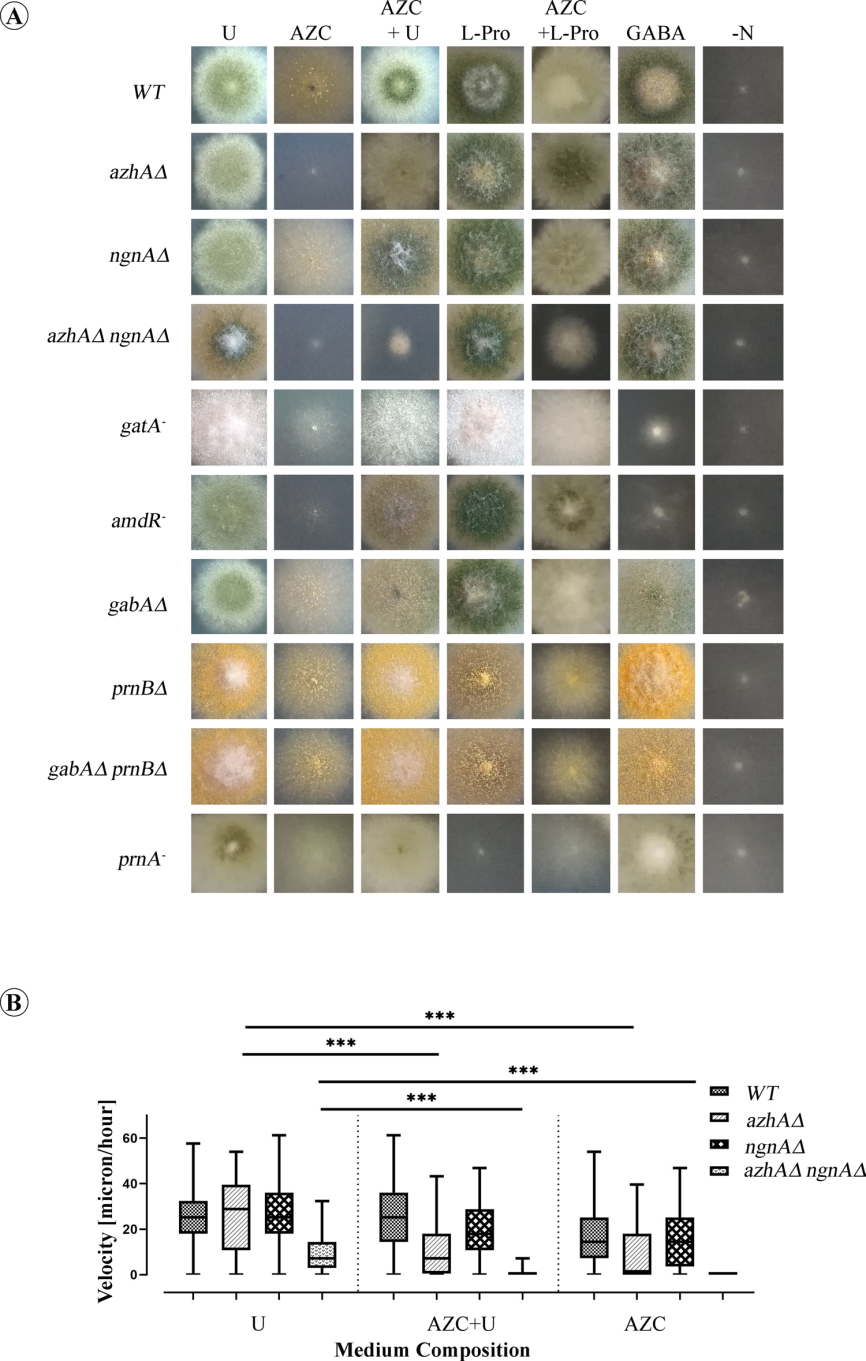
AzhA is required for growth of *A. nidulans* in the presence of AZC. (**A**) Growth of WT, *azhA*Δ*, ngnA*Δ*, azhA*Δ *ngnA*Δ*, gatA*^*-*^*, amdR*^*-*^*, gabA*Δ*, prnB*Δ*, gabA*Δ *prnB*Δ *and prnA*^-^ *A. nidulans* strains on MM with Urea (U); AZC; GABA and L-Pro, as nitrogen sources at a final concentration of 5 mM, or on MM lacking any nitrogen source (–N). Growth of *ngnA*Δ single deletion strain in the presence of AZC either as sole nitrogen source (AZC) or with urea (AZC + U) is similar to that of the WT strain. On the other hand, growth of *azhA*Δ single deletion strain in the presence of AZC as sole nitrogen is severely impaired, while in AZC + U is similar to that of the WT, indicating that AzhA is involved in AZC assimilation. Accordingly, growth of the *ngnA*Δ *azhA*Δ double deletion strain is severely impaired in both AZC or in AZC + U, indicating the involvement of the corresponding enzymatic activities in AZC detoxification and assimilation. Growth of *gatA*^-^ and *amdR*^-^ strains on AZC as sole nitrogen source is highly reduced compared to the WT strain, supporting that AZC is assimilated through the GABA metabolic pathway. On the contrary, growth of *prnB*Δ and *prnA*^*-*^ strains on AZC as sole nitrogen source is similar to that of the WT strain, supporting that AZC is not assimilated through the proline catabolic pathway. Finally, *gabA*Δ*, prnB*Δ*, gabA*Δ *prnB*Δ mutant strains show no growth defect in the presence of AZC as sole nitrogen source, suggesting that AZC is not transported through the major proline transporter PrnB or the GABA transporter GabA. (**B**) Growth rate of *A. nidulans* WT, *azhA*Δ*, ngnA*Δ and *azhA*Δ *ngnA*Δ germlings in submerged liquid cultures, using (U), (AZC + U) or AZC as nitrogen sources. Liquid media were inoculated with conidiospores and incubated for 18 h at 25 °C. Hyphal growth rate was observed at frames taken every 15 min for a total of 45 min. Values are plotted in box-and-whiskers plots. *Asterisks* indicate a significant difference of the mean (*P* ≤ 0.05) between two conditions. Statistical significance was analyzed via the Kruskal–Wallis Test and is depicted with asterisks (*). Single (*), double (**) or triple (***) asterisks, indicate 0.01 ≤ p < 0.05, 0.001 < P ≤ 0.01, or p < 0.001, respectively.

Consistently with our previous results^25^, we confirmed that AZC is not toxic to *A. nidulans*. In this study, we further demonstrated that AZC can be utilized as a sole nitrogen source (Fig. 1). Therefore, given that growth with AZC as sole nitrogen source is NgnA-independent (Fig. 1), we investigated whether an AZC-hydrolase, similar to the AZC-assimilating hydrolase of *Pseudomonas* A2C strain (AC-hydrolase), is present in *A. nidulans*. The Basic Local Alignment Search Tool for proteins (BLASTp), and the amino acid sequence of *Pseudomonas* AC-hydrolase as query (Fig. S1 in supplementary material), were used to retrieve sequences with strong similarity in *A. nidulans*. The results obtained, indicated the product of the AN12472 gene (designated also as AN9518) as a top-scoring match, with 44.54% percentage identity and 97% query coverage. AN12472 is an uncharacterized gene, with an intronless Open Reading Frame (ORF) of 726 bp, encoding a 241 amino acid polypeptide with a predicted hydrolase activity, acting on acid halide bonds. This amino acid sequence was designated AzhA (**Αz**etidine **h**ydrolase). Deletion of the *azhA* gene resulted in the inability of the corresponding strain to utilize AZC, as evidenced by a noticeable decrease in the growth of the *azhA*Δ deletion strain in the presence of AZC as sole nitrogen source (Fig. 1A). In support of this, AZC is slightly toxic to *azhA*Δ in the presence of urea (Fig. 1A,B). As previously reported, deletion of the *ngnA* gene (encoding an MPR1/2 orthologue), does not confer AZC sensitivity to *A. nidulans*^25^. Moreover, *ngnA*Δ deletion strain macroscopically grows similarly to a wild-type (WT) strain in the presence of AZC as sole nitrogen source. Hence, we examined the growth of the *azhA*Δ *ngnA*Δ double deletion strain, in comparison to the WT and the corresponding single deletion strains, *azhA*Δ and *ngnA*Δ. As documented in Fig. 1A, growth of the *azhA*Δ *ngnA*Δ strain is severely impaired by the presence of AZC, when urea is used as a nitrogen source, supporting the involvement of both enzymes in AZC detoxification.

As a complementary to the above approach, we measured the hyphal growth rate of several strains in the presence or absence of AZC, in submerged liquid cultures, using confocal microscopy. The results (Fig. 1B, see also Video S1 in supplementary material) show that WT hyphae from germlings that have grown for 18 h with urea as sole nitrogen source, have an apical growth rate of 25.89 ± 10.38 μm/h. This rate is nearly identical (26.12 ± 14.96 μm/h) to that of germlings incubated in the simultaneous presence of urea and AZC (U + AZC), further confirming that AZC is not toxic to *A. nidulans*. Accordingly, *azhA*Δ and *ngnA*Δ single deletion strains have almost similar mean values of growth rate, when grown in urea. Interestingly, we noticed that the growth rate of the *azhA*Δ *ngnA*Δ double mutant germlings with urea as sole nitrogen source was statistically significantly lower compared to either the WT or the single deletion strains. This observation was also evident when different nitrogen sources were used, e.g. ammonium and glutamate (data not shown), suggesting that both enzymes might somehow be implicated in the hyphal polar growth or germination of conidiospores. As expected, the growth rate of the WT strain was reduced in the presence of AZC as the sole nitrogen source, with a mean growth velocity of 16.91 ± 13.97 μm/h, confirming that AZC is a poor nitrogen source for *A. nidulans*. On the contrary, the growth rate of the *azhA*Δ deletion strain significantly decreased when both AZC and urea were present in the growth medium. Growth of the *azhA*Δ *ngnA*Δ double mutant was, as expected, severely inhibited when AZC was supplemented as sole nitrogen source, in agreement with growth tests in solid media. When both urea and AZC were supplemented, only approximately 20% of cells accomplished emergence of the germ tube, indicating the severe toxicity of AZC in the double deletion strain (Fig. 1B).

In addition, it was observed that *ngnAΔ* single deletion strain in U + AZC had a growth rate 1.5 fold lower than in urea (U) alone. This phenotype of the *ngnAΔ* strain, which was not clearly observed with the conventional solid media growth tests, indicates an important contribution of the NgnA acetyltransferase in the detoxification of AZC in germlings. In total, the microscopic approach used for growth observations^29,30^, enabled us to measure more clearly the polar growth rate of our strains grown with different nitrogen sources and to further confirm that both AzhA and NgnA proteins contribute to AZC detoxification, while only AzhA is required for the utilization of AZC as a nitrogen source in *A. nidulans*.

### Catabolism of AZC in *A. nidulans* is AmdR/IntA- and GABA-dependent and L-proline independent

In order to gain insights into the metabolic pathway of AZC catabolism in *A. nidulans*, and given that it is a proline analogue whose detoxified hydrolysis product in *Pseudomonas* is transported into the cytoplasm by the action of a GABA transporter homologue (see above), we examined AZC transport in strains defective for proline (*prnB*Δ) and γ-aminobutyric acid (GABA) (*gabA*Δ*)* uptake, as well as in strains defective in the GABA (*amdR*^*-*^*, gatA*^*-*^) or L-Pro (*prnA*^-^) metabolic pathways. The *prnB*Δ strain carries a deletion in the gene of the main proline transporter, PrnB^31,32^, and accordingly the *gabA*Δ carries a deletion in the gene coding for a GABA permease^25,33^. Strains designated as *gatA*^*-*^ and *amdR*^*-*^ carry loss-of-function mutations in the genes expressing the GABA transaminase (GatA), and its positively acting regulator AmdR/IntA, respectively^26,27,34^. Finally, the *prnA*^*-*^ strain carries a loss-of-function mutation in the gene expressing the specific positively acting regulator of the five clustered structural genes of L-Pro uptake and catabolism^35,36^. Growth tests presented in Fig. 1A show that, in the presence of AZC as sole nitrogen source, both *gatA*^*-*^ and *amdR*^*-*^ loss-of-function strains have severely reduced growth compared to WT, similar to that of the *azhA*Δ strain. This reduction in growth is probably due to the inability of the *gatA*^*-*^ and *amdR*^*-*^ strains to sufficiently assimilate AZC via the GABA metabolic pathway, rather than AZC as a sole nitrogen source itself being toxic, since growth of both strains in AZC + U is similar to that of the WT strain. On the other hand, strains lacking either the main L-Pro (*prnB*Δ*)*, or the main γ-aminobutyric (*gabA*Δ) transporters or both (*prnB*Δ *gabA*Δ), show no growth defect in the presence of AZC as sole nitrogen source (Fig. 1A), suggesting the existence of other transporter(s) responsible for AZC uptake. This transporter is not the product of AN5678, the strict homologue of the general amino acid permease Gap1 of *S. cerevisiae*^37^, since the triple deletion strain lacking all permeases is still able to utilize AZC (data not shown). Furthermore, the *prnA*^*-*^ loss-of-function strain shows similar growth to the WT in the presence of AZC, indicating that AZC is not catabolized via the L-Pro catabolic pathway (Fig. 1A).

### Expression of *azhA* and *ngnA* is induced by AZC

**Figure 2:**
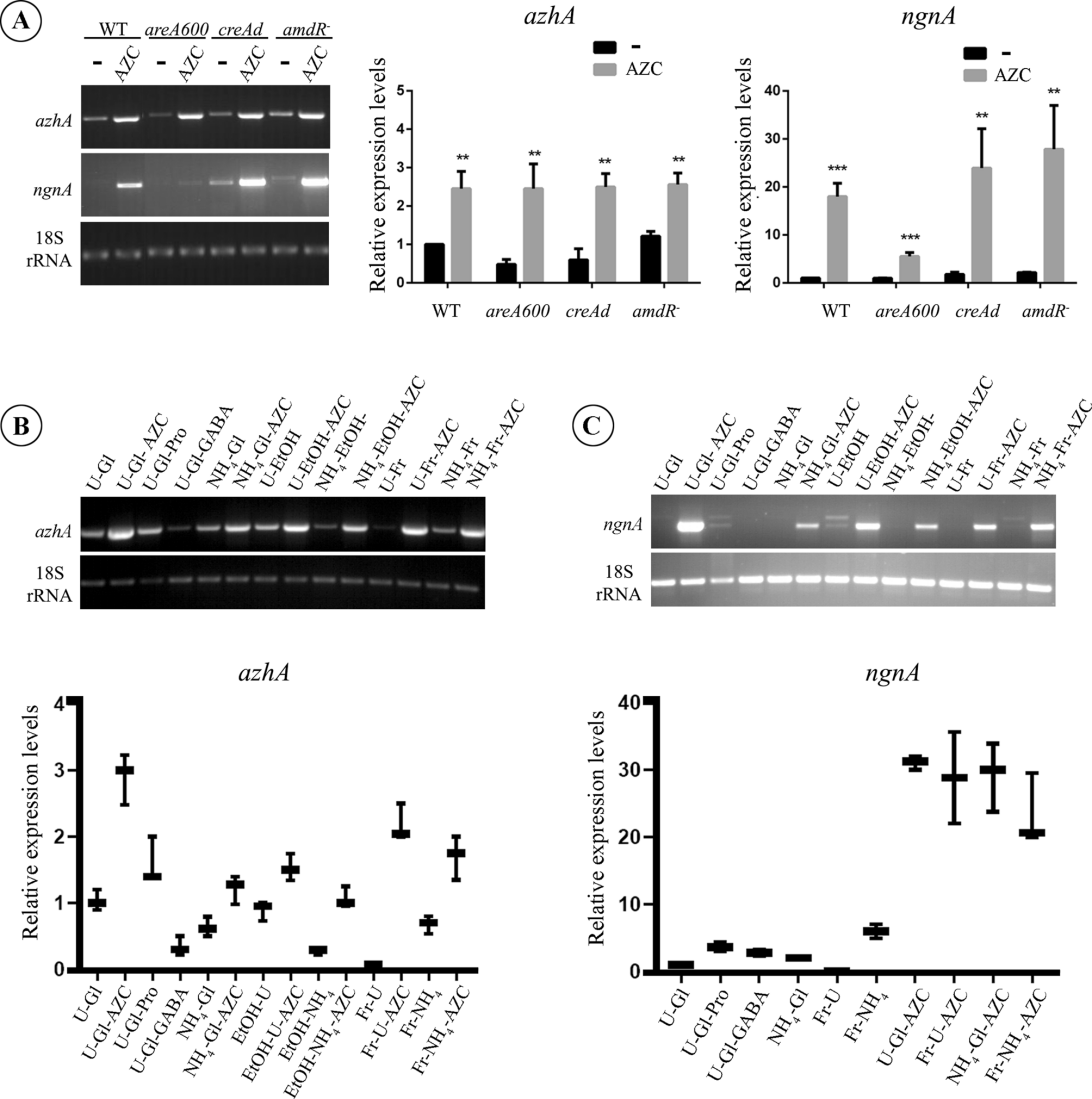
Expression of *azhA* and *ngnA* genes in different strains and growth conditions. Total RNA was extracted from hyphae of WT, *areA600*, *creA*^*d*^ and* amdR*^-^, strains on appropriately supplemented MM and incubated at 25 °C. (**A**) Representative images of RT-PCR-derived mRNA transcripts of *azhA* and *ngnA* genes in the presence of urea (-) or urea and AZC (AZC) as nitrogen sources and glucose as carbon source. (**B** and **C**) Effects of L-Proline (Pro), GABA or ammonium (NH^+^_4_) as nitrogen sources and glucose (Gl), fructose (Fr) or ethanol (EtOH) as carbon sources, on the expression of the *azhA* (**B**) and *ngnA* (**C**) genes, respectively in a WT strain. Levels of *azhA* and *ngnA* transcripts in different conditions were normalized by 18S rRNA levels. Statistical significance was analyzed via two-way ANOVA and is depicted with asterisks (*). Single (*), double (**) or triple (***) asterisks, indicate 0.01 ≤ p < 0.05, 0.001 < P ≤ 0.01, or p < 0.001, respectively.

To our knowledge, there is little information on the regulation of expression of genes involved in resistance to AZC in *S. cerevisiae*, *Pseudomonas* or their homologues in plants^15,38^. Expression of *A. nidulans* genes encoding proteins involved in the uptake and catabolism of nitrogenous compounds is mainly regulated at the transcriptional level and is induced only when their substrates are available^34–35^. Therefore, we examined whether the expression of the *azhA* and *ngnA* genes is subject to transcriptional regulation by AZC, using RT-PCR. RNAs were extracted from a WT strain grown with urea (U) as a nitrogen source in the presence or absence of AZC. Our results show an approximately four-fold increase of *azhA* (Fig. 2A) and more than a 15-fold increase of *ngnA* transcript levels in the presence of AZC (Fig. 2A), indicating that AZC acts as a potent inducer of the genes involved in AZC detoxification and/or catabolism. Interestingly, we have observed that *ngnA* basal expression levels were quite low in non-induced conditions, and we could detect a slow migrating band corresponding to unspliced RNA (pre-mRNA), which we confirmed was not due to DNA contamination (see Materials and Methods). In the presence of AZC this band disappeared, suggesting an AZC-dependent induction in the splicing of the *ngnA* gene (investigation in progress).

Since AZC is an L-Pro analogue, we further investigated the involvement of L-Pro in the regulation of *ngnA* and *azhA* gene expression. It is well established that *A. nidulans* is able to utilize L-Pro as sole nitrogen and carbon source using enzymes mapped in five genes clustered in linkage group VII (*prn* cluster). The structural genes of the *prn* cluster are not transcribed in the absence of L-Pro, or in a genetic background lacking the cluster specific positive regulatory protein, PrnA^41^. Our results presented in Fig. 2B and C show that L-Pro does not induce the expression of either *azhA* or *ngnA* genes. Along with that, absence of PrnA does not affect either the utilization of AZC as poor nitrogen source or its toxicity, as evidenced by the similar growth of a *prnA*^-^ loss-of-function and the WT strain in the presence of AZC (Fig. 1A).

In *A. nidulans,* the enzymes involved in the utilization of nitrogenous sources are subject to nitrogen metabolite repression (NMR), mediated by the general transcriptional activator AreA^34,36^. Thus, as AZC can be utilized as a poor nitrogen source (Fig. 1A), we have examined whether *azhA* and *ngnA* expression is regulated by AreA. Towards this, the expression of both genes was investigated in an *areA* null mutant strain (*areA600*) and compared to that of the WT strain, using RT-PCR. An *areA600* strain favors ammonium as nitrogen source for its growth, while it shows a moderate growth with urea and a complete lack of growth with any other secondary nitrogen source, like L-Pro or nitrate^42^. Our results presented in Fig. 2A, show that in the presence of AZC, *ngnA* expression is reduced in *areA600* compared to the WT strain, suggesting an AreA-dependent *ngnA* expression. However, AZC-induced *ngnA* expression is not affected by the presence of ammonium (Fig. 2C), a condition well-known to inactivate AreA^36,43^. Therefore, it appears that reduced induction of *ngnA* in *areA600* is most probably due to an ammonium independent AreA regulation (see discussion). On the other hand, transcript levels of *azhA* remain approximately similar in WT and *areA600* strains, suggesting an AreA-independent regulation of *azhA* expression (Fig. 2B)*.* Interestingly, in an *azhAΔ ngnAΔ* double mutant strain ammonium partially protects from AZC toxicity (data not shown), suggesting an AreA-dependent expression of the AZC-transporter. This might in turn explain the small reduction observed in the AZC-induced levels of both the *azhA* and *ngnA* genes in the presence of ammonium.

In *A. nidulans,* the enzymes involved in the utilization of carbon sources, including L-Pro, are subject to carbon catabolite repression (CCR), mediated by the general transcriptional suppressor CreA. Therefore, as AZC is an L-Pro analogue, we have examined whether *azhA* and *ngnA* expression is regulated by CreA. Towards this, the expression of both genes was investigated in a constitutive carbon-catabolite-derepressed strain *(creA*^*d*^)^44,45^, by RT-PCR. As shown in Fig. 2A, *azhA* transcript levels are similar in *creA*^*d*^ and WT strains, in both the presence and absence of AZC. On the contrary, *ngnA* transcript levels are slightly increased in *creA*^*d*^ compared to the WT (Fig. 2A), in an AZC-independent manner. However, the basal levels of *ngnA* expression or the levels of its AZC-mediated induction are not increased when ethanol or fructose instead of glucose is utilized as a sole carbon source (Fig. 2B,C). As it is well established, CreA is inactive when a secondary carbon source like ethanol or fructose is utilized^36^.Thus, *ngnA* regulation by CreA is repressing carbon source independent.

Our data suggest that AZC is catabolised in *A. nidulans* through the GABA metabolic pathway (Fig. 1A). Hence, we investigated whether *azhA* and *ngnA* expression is regulated by GABA and/or AmdR/IntA, the positive acting specific transcription factor regulating the expression of five structural genes involved in acetamide (*amdS*), omega amino acid (*gatA* and *gabA*) and lactam (*lamA* and *lamB*) catabolism^26,27,46^. Firstly, transcript levels of both *azhA* and *ngnΑ* genes were examined, as above, in an *amdR*^*-*^ null mutant strain. As shown in Fig. 2A, induction of *azhA* is similar in *amdR*^*-*^ and WT strains. On the other hand, *ngnA* transcript levels were increased in the *amdR*^*-*^ strain in the presence of AZC, suggesting that either AmdR/IntA acts as a transcriptional repressor of *ngnA* expression, or that a potential over-accumulation of AZC in the *amdR*^*-*^ strain, due to lack of AZC assimilation, leads to overexpression of *ngnΑ*. The latter scenario would suggest that AZC itself, and not GABA is the inducer of *azhA* and *ngnA* expression. In order to investigate this possibility, we examined whether GABA acts as an inducer of *azhA* and *ngnA* expression. As documented in Fig. 2B,C, GABA does not induce either *azhA* or *ngnA* expression. Therefore, we conclude that AZC itself is the inducer of *ngnA* and *azhA* expression.

**Figure 3:**
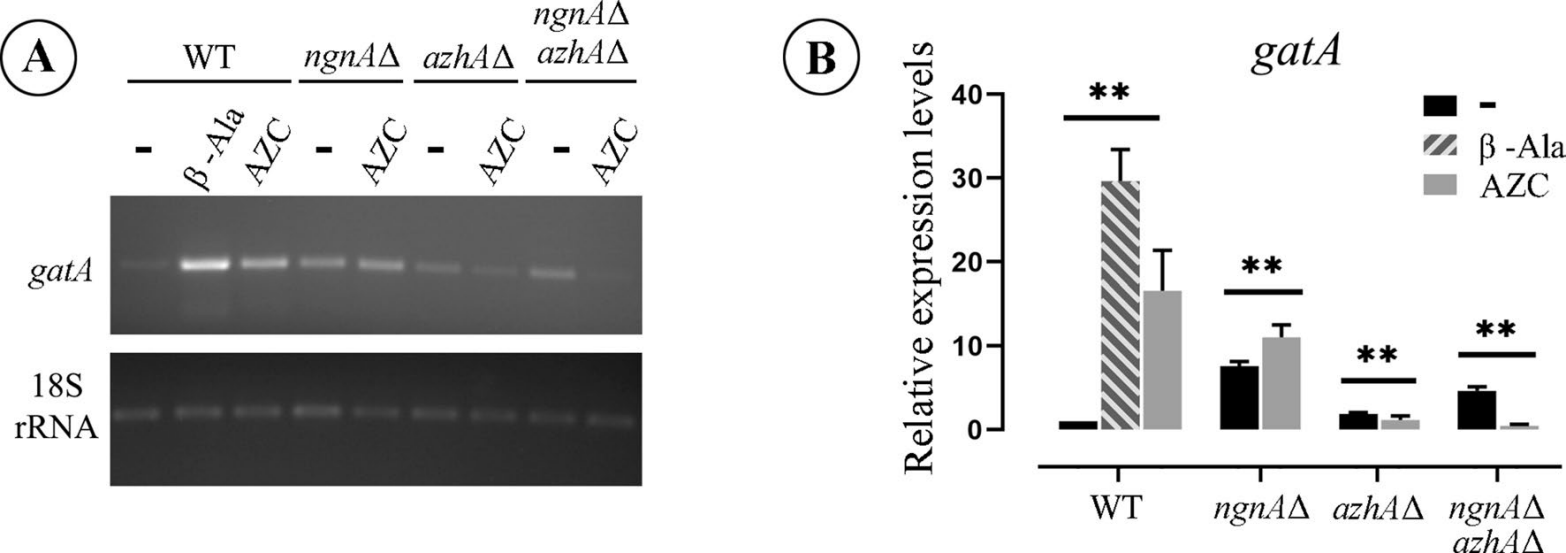
Expression of *gatA* under AZC induction. Total RNA was extracted from hyphae of WT, *azhA*Δ*, ngnA*Δ and *azhA*Δ *ngnA*Δ strains on appropriately supplemented MM incubated at 37 °C. (**A**) Representative image of RT-PCR-derived mRNA transcripts of *gatA* gene in non-inducible condition (–) and in the presence of β-alanine (β-ala) or AZC, and fructose (Fr) as carbon source. (**B**) Relative quantification and statistical analysis of *gatA* transcripts in non-induced condition (–), β-ala or AZC, normalized by 18S rRNA levels. Statistical significance was analyzed via two-way ANOVA and is depicted with asterisks (*). Single (*), double (**) or triple (***) asterisks, indicate 0.01 ≤ p < 0.05, 0.001 < P ≤ 0.01, or p < 0.001, respectively.

The above results are consistent with the possibility that AZC is converted to GABA in order to be assimilated. To examine this, we investigated whether AZC induces the expression of the *gatA* GABA transaminase gene, well-known to be induced by GABA and β-alanine^27,33^. As documented in Fig. 3A,B in the WT strain, *gatA* expression is induced in the presence of AZC in a way similar to the presence of β-alanine, its natural inducer^40^. Most importantly, this induction requires a wild-type *azhA* gene. These results suggest that upon AZC feeding and only in the presence of AzhA, GABA is produced and induces the expression of *gatA,* strongly indicating that AZC is metabolized through the GABA pathway via the action of the AzhA hydrolase.

In total, our results show that AZC is not catabolized through the L-Proline metabolic pathway, and it rather requires an intact GABA pathway. Moreover, AZC-mediated induction of both *ngnA* and *azhA* genes suggest an AmdR/IntA- and GABA-independent mechanism of their regulation at the transcriptional level (see also discussion).

### Heterologous expression of AzhA hydrolase in *S. cerevisiae*

**Figure 4:**
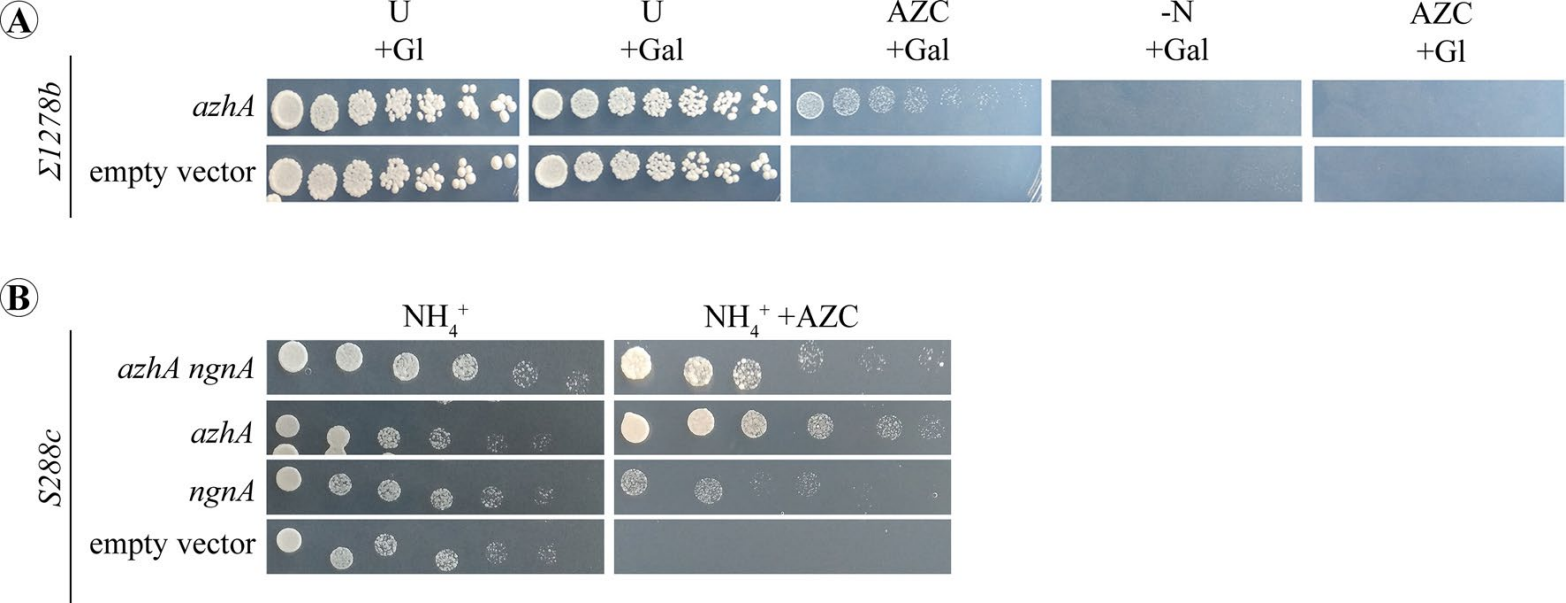
Growth assays of *S. cerevisiae* strains expressing the *A. nidulans azhA* and *ngnA* ORFs. (**A**) Serial dilution assays of Σ1278b strains expressing either the *azhA* ORF under the regulation of the Gal1 glucose-repressible promoter, or the corresponding empty vector. The Σ1278b strain expressing the *azhA* ORF (*azhA*) is able to utilize AZC as a nitrogen source only in the presence of galactose (Gal) as a carbon source (AZC/Gal). (**B**) Serial dilution assays of S288C strains expressing either the *A. nidulans* the *azhA* and / or the *ngnΑ* ORFs under the regulation of the Gal1 promoter, or the corresponding empty vector. The S288C strain expressing either the *azhA* or the *ngnΑ* ORF is able to detoxify AZC.

Τhe Σ1278b strain of *S. cerevisiae* containing the gene encoding the acetyltransferases Mpr1/2, orthologues of the NgnΑ acetyltransferase in *A. nidulans*, is resistant to AZC, but unable to utilize AZC as a nitrogen source^19^. Therefore, heterologous expression of AzhA in Σ1278b was used to confirm that AzhA hydrolase is necessary and sufficient for AZC-assimilation, since this strain is known to be able to use GABA as a nitrogen source^48^. Towards that, Σ1278b was transformed individually with a plasmid containing the *azhA* gene expressed under the *gal1* promoter, which is activated in the presence of galactose^49^, and with an empty vector as a control (Tables 2 and 3). Our results show that only upon induction of AzhA expression (galactose as carbon source), Σ1278b strain becomes able to utilize AZC as a nitrogen source (Fig. 4A). It is additionally known that the widely used strain of *S. cerevisiae*, S288C, does not possess the Mpr1/2 acetyltransferases, and is therefore AZC-sensitive^18^. We thus used this strain to complement the lack of Mpr1/2 by heterologous expression of *A. nidulans* NgnΑ acetyltransferase (Fig. 4B). Using the approach described above, the S288C strain was transformed separately, with plasmids expressing *ngnΑ* and/or *azhA* genes, under the control of the *gal1* promoter, and with an empty vector as a control (Tables 2 and 3). Our results are consistent with NgnA being an orthologue of Mpr1 and thus conferring the ability to the S288C strain to detoxify AZC (Fig. 4B).

In total, our results show that AzhA hydrolase is necessary and sufficient for the detoxification of AZC and its utilization as a nitrogen source in *A. nidulans*, most probably via the GABA assimilation pathway, while NgnΑ acetyltransferase additionally contributes to the detoxification of AZC.

### Homologues of AzhA are scarcely found among bacterial and fungal species

To investigate whether proteins homologous to AzhA exist in species belonging to the three domains of life (Archaea, Bacteria and Eukaryotes) we searched the Database of National Center for Biotechnology Information (NCBI) with BLASTp, using the AzhA amino acid sequence, as query. The parameters of our searches were at least 80% coverage of the query sequence and E-values lower than 1e^-05^. Multiple sequence alignment indicated the conservation of the four highly conserved sequence motifs by which HAD-family members are recognized (in detail,—FDxDG,—T/SXx,—KPxP, and ED or GDxxxDD)^28,47–52^ among the fungal species that were retrieved by our BLASTp search (Supplementary Figure. S2) and were used for our phylogenetic analysis.

Moreover, sequence similarity to AzhA was found in all three major phylogenetic domains—i.e. Archaea, Bacteria and Eukaryotes (see Table S3 in the supplementary material). In eukaryotes, homologues of AzhA are mainly found in specific fungal taxa. Surprisingly enough, in the remaining eukaryotes, AzhA homologues were retrieved as single hits in species belonging to the lower eukaryotes (plakozoan, *Trichoplax adhaerens*) and to the kingdoms of Plantae (*Quercus suber*) and Metazoa (lepidopteron, *Eumeta japonica*).

Within the kingdom of Fungi AzhA, homologues are mainly found in species of the Eurotiales Order. On the contrary, both homologues of the amino or nucleic acid sequences of AzhA are missing from the genomes of the Saccharomycotina subphylum and the early diverging fungal lineages, such as Microsporidia, Chytridiomycota, Glomeromycota or Zygomycota^53^. Τhe only exception was in the subphylum of Mucomycotina, where only five hits were found. The amino acid matrix of AzhA homologues produced phylogenetic trees, which were almost invariant to the method applied (see Fig. S3 and S4 in the supplementary material).

**Figure 5:**
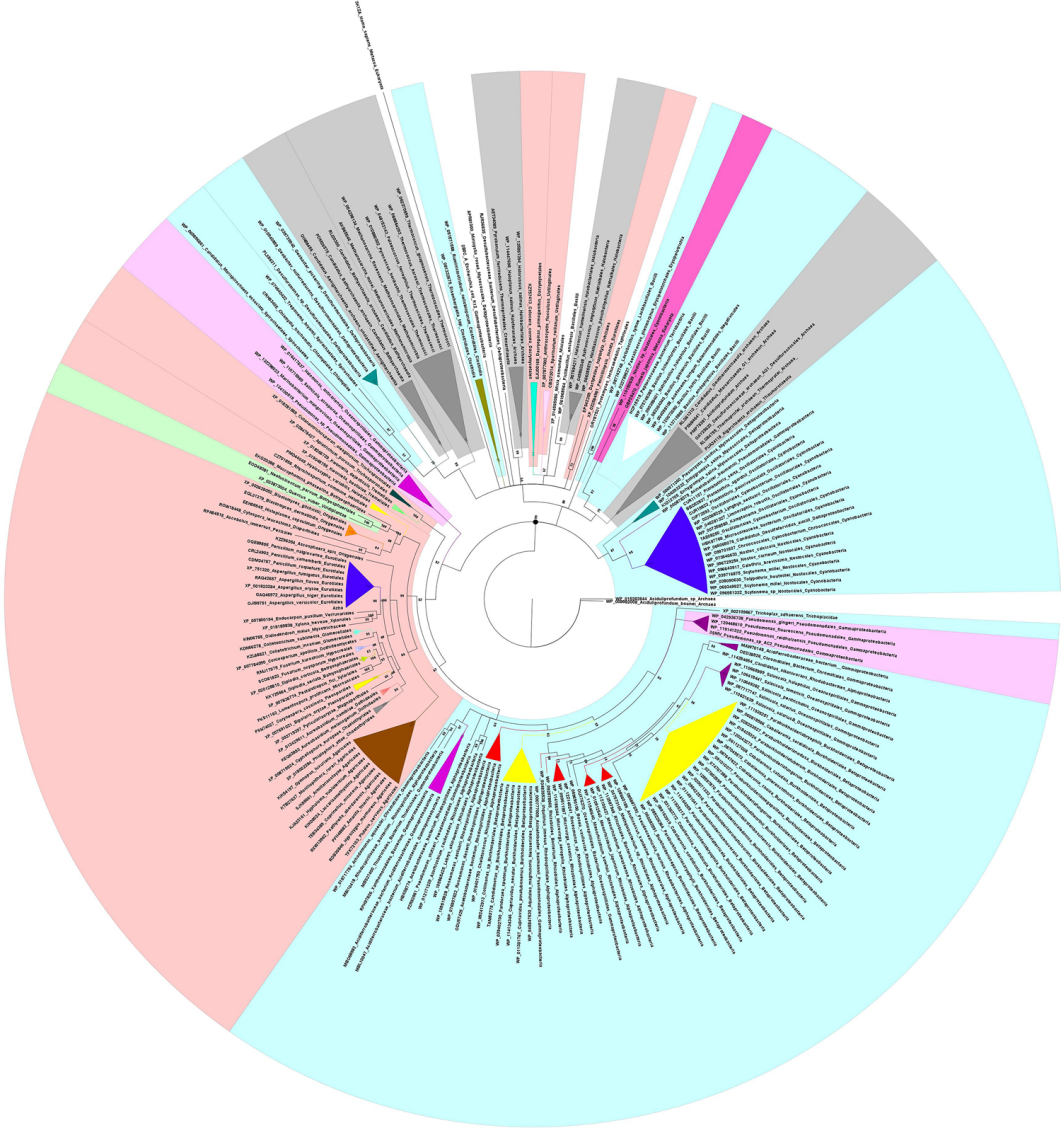
Phylogenetic relations of AzhA homologues in the three domains of life (Archaea, Bacteria and Eukaryotes), as emerged by the implementation of a MCMC tree, under the evolutionary model LG + G + F. All topologies produced are in agreement with the NJ analysis used as an alternative method. The tree is divided into two big clusters, which are noted with a black circle on the node that discriminates them. The coloring of the highlighted species is as follows: blue, green, grey and pink for bacteria, plants, archaea and fungi, respectively. The *Aciduliprofundum spp*. was used to root the tree. Tree visualization was conducted with Figtree.

All of our data were included in a “Universal tree”, in which, AzhA homologues are shown to split into two main clusters (Fig. 5). The first one consists of proteins mostly from Archaea, several Classes of Bacteria (i.e., Cyanobacteria, Delta-proteobacteria, Bacilli, Negativicutes, Erycipelotrichia, Clostridia, Spirochaetia and Halobacteria), and a few homologues from fungal classes which cannot be found in the second cluster (Dacrymycetales, Mixiales, Mucorales, Orbiliales, Taphrinales and Ustilaginales). The second cluster consists of Bacteria belonging to the classes of Alpha-, Beta- and Gamma- proteobacteria, Fungi from the phyla of Basidiomycota and Ascomycota (with the exception of species belonging to Cluster 1) and the remaining few representatives of Eukaryota (Fig. 5). In Ascomycota most results are found in the leotiomyceta clade, which are divided among the classes of Dothideomycetes, Eurotiomycetes and Sordariomycetes. Accordingly, in the phylum of Basidiomycota most results are found in the classes of Agaricomycetes and Tremellomycetes. The discrimination of these two clusters is strongly supported by both methods used (PP: 99% and NJ-bootstrap: 92.65%).

The *A. nidulans* AzhA protein examined in this work, is placed within the Eurotiales clade with excellent support (PP: 100% and NJ-bootstrap: 94.33%) as expected (see Fig. S3 and S4 in the supplementary material). Another interesting aspect of the topology of the tree is that the basal group of the second cluster contains *Pseudomonas* and other relative genera, all belonging to Gamma-proteobacteria.

In addition, we noticed the strong clustering (100% support with both statistical methods used) of the homologue belonging to the sole plant representative (*Quercus suber*) in the tree with the putative homologue sequence of *Neofusicoccum parvum*, an aggressive phytopathogen, member of the Botryosphaeriaceae^51–56^. The close clustering of these two species could possibly indicate a gene transfer event between a plant and a plant pathogen. Accordingly, our attention was drawn by the presence of a putative AzhA homologue in a *Candidatus* Colwellia aromaticivorans sp. (Fig. 5). Interestingly, it was recently reported that strains of *Candidatus* Colwellia aromaticivorans sp. can be used to degrade sea oil spills^57^. Overall, these sequence data could provide good evidence for the identification of horizontal gene transfer (HGT) events between plants and fungal phytopathogens, as it has been previously shown^58^.

## Discussion

*A. nidulans* as a soil fungus^59^ could potentially share the same ecological niches with AZC producing plants. AZC possibly serves as an allelochemical among plants, fungi and bacteria, maintaining unknown balances among them, like several other plant secondary metabolites do^60,61^. In this work we showed that *A. nidulans* possesses a specific, dual mechanism in order to cope with AZC toxicity, but also to utilize it as a nitrogen source. This detoxification mechanism relies on the presence of a hydrolase, designated AzhA, and an auxiliary acetyltransferase, NgnA^25^. In the presence of AZC as a sole nitrogen source, *azhA*Δ *ngnA*Δ double deletion strain shows no residual growth in both solid and liquid media, reflecting the highly toxic effect of AZC, when intracellularly accumulating. Specifically, our microscopic observations showed an 80% inhibition of *azhA*Δ *ngnΑ*Δ spore germination in the presence of AZC, a phenotype similar to that observed macroscopically in solid growth tests (see Fig. 1B; also see Supplementary Video S1 in the supplementary material, online).

Our phylogenetic analysis indicated the high conservation of AzhA sequence among several taxa of fungi and gamma-proteobacteria. Collectively, our results strongly suggest that AzhA is a true orthologue of the A2C *Pseudomonas* hydrolase^22^, and is responsible for AZC detoxification and assimilation in *A. nidulans*. Thus, it is highly probable that the AzhA enzyme of *A. nidulans* functions similarly to its *Pseudomonas spp.* homologue and converts AZC to a GABA-like product, which could subsequently be assimilated via the GABA utilization pathway. Indeed, our results indicate that utilization of AZC as a source of nitrogen is independent of the L-Pro catabolic pathway and rather requires an intact and functional GABA catabolic pathway. More specifically, we show that the presence of AZC induces the expression of the GABA-inducible GatA GABA transaminase, and this requires an intact *azhA* gene, strongly suggesting that AzhA hydrolyses AZC to a GABA-like intermediate. The latter would promote the induction of *gatA* expression. We also show that heterologous expression of *azhA* in different *S. cerevisiae* strains is sufficient for AZC detoxification. Since these yeast strains are known to be able to use GABA as a sole nitrogen source^62^, this result shows that the sole reason for the lack of AZC utilization in yeasts is the lack of an AZC hydrolase, and is yet another indication that AzhA converts AZC to a GABA-like intermediate. It is also noteworthy that, contrary to *S. cerevisiae*, AZC uptake in *A. nidulans* is not mediated via neither the major L-Pro or GABA transporters, PrnB or GabA respectively, nor via the product of AN5678, the closest homologue of the General amino-acid permease Gap1^37^. More work is needed in order to identify the AZC transport systems of the fungus, whose expression and activity seems to be, at least partially, nitrogen metabolite repression-dependent and carbon catabolite repression-independent. Our results support the scenario that AZC itself acts as the inducer for the expression of both *azhA* and *ngnA* genes*.* More specifically, AZC is still able to induce the expression of *ngnA* in the *azhA*^*-*^ strain that is unable to metabolize AZC (Fig. 2), showing that conversion of AZC to a GABA-like intermediate is not required for the induction of *ngnA*. In addition, *ngnA* and *azhA* expression does not depend on, AmdR/IntA, the specific transcriptional activator of the GABA catabolic pathway genes. In fact, AZC causes an over-induction of *ngnΑ* in the *amdR*^-^ strain. Given that this strain is unable to metabolize GABA, the above suggest that intracellular overaccumulation of AZC or GABA leads to increased expression of the AZC-detoxifying enzymes. GABA supplementation, however, does not induce the expression of *ngnΑ* and *azhA* (Fig. 2), strongly indicating that the reason for *ngnΑ* over-expression in the *amdR*^-^ strain should be AZC over-accumulation. Importantly, expression of *ngnA* does not directly depend on either AreA or CreA general transcription factors either, as evidenced by similar basal and AZC-dependent induced levels of *ngnΑ* expression in the wild-type strain under both nitrogen (NH_4_^+^) and carbon (glucose; Gl) repressed conditions of growth compared to derepressed conditions (urea and fructose, respectively). Similarly, *azhA* expression is AreA and CreA independent (Fig. 2A,B). Thus, transcriptional regulation of *ngnΑ* and *azhA* expression differs from the usual situation of enzymes involved in the assimilation of secondary carbon and nitrogen sources in *A. nidulans*, that are under nitrogen metabolite repression and carbon catabolite repression^36,63,64^. An interesting explanation for this difference would be that the mechanism of *ngnΑ* and *azhA* induction is evolutionarily adapted to confer protection from AZC toxicity under all conditions of nutrient availability, given that AZC import into the cells seems to be mediated by transport systems that are also independent of nitrogen metabolite repression and carbon catabolite repression (see above).

Interestingly, we found *ngnA* basal expression levels to be quite low in non-induced conditions, and a slow migrating band in the absence of AZC, most probably corresponding to unspliced mRNA, is observed. In the presence of AZC this band disappears, suggesting an AZC-dependent splicing of *ngnA* upon its AZC-mediated induction. This observation is reminiscent of a recent report in mammalian cells, where intron retention of several transcripts was shown to correlate with nuclear localization of the mRNA^65^. It would, thus, be tempting to speculate that the splicing and nuclear export of the *ngnA* mRNA could be promoted, via an unknown mechanism, by the presence of its own substrate.

Our work provides evidence that AzhA is a member of the HAD superfamily, which encompasses various enzymes that catalyze carbon or phosphoryl group transfer reactions on a diverse range of substrates, using an active site of aspartate in nucleophilic catalysis^50^. Members of this enzyme superfamily have been shown to participate in the detoxification of xenobiotics and several other toxic substances^28^, being in agreement with our results suggesting AzhA as a detoxifying enzyme. Moreover, recent reports have proposed that some members of this family are genetically associated with human diseases such as cancer, cardiovascular, metabolic and neurological disorders^66^. In this work we employed a sequence-based phylogenetic analysis in order to explore the evolutionary history of AzhA. As expected, AzhA is located into the group of homologues belonging to Eurotiales. However, this phylogenetic relation was restricted to the taxonomic level of the Order and in some cases to the level of Class. This result is in contrast with single gene phylogenies of housekeeping genes, like beta-tubulin^67^ or the mitochondrial *rps*3^68^, where the evolution of the examined sequences coincides with the evolution of the organism carrying them. This is expected since single gene phylogenies do not represent the evolutionary history of the whole organism but only of the gene itself^69,70^. Moreover, our phylogenetic results indicate that AzhA has homologues in several fungal, bacterial and archaeal species. We noticed the high sequence conservation of AzhA amongst certain taxa in the kingdom of fungi, like the genera of Aspergilli, Penicillia and the order of Agaricales. On the contrary, we also noticed the complete absence of homologues in several other taxa, like the subdivision of Saccharomycotina. The most plausible interpretation of these results would be the presence of an AzhA homologue before the division of Dikarya, probably in the last fungal common ancestor; and afterwards, a series of several horizontal gene transfers (HGT) and losses throughout its evolution, which led to the corresponding presence or absence to the several fungal taxa. The relative posed question would be how this ancestral AzhA homologue “reached” the last common fungal ancestor, was it a case of horizontal gene transfer or was an endosymbiotic gene transfer event (EGT)? The presence of putative AzhA homologues in several Archaea species could provide some theoretical indications for a probable EGT event, even before the genesis of the last fungal common ancestor.

The most widely accepted endosymbiotic theory of the LECA^71^, supports the notion that the nuclear genome of eukaryotes derives from an ancestral state of a chimeric Archaeon^72,73^. From our analysis, this is reflected in the case of AzhA from the clustering of the early diverging fungal homologues (i.e. from Mucoromycotina) and the few other fungal homologues with the Archaea (Fig. 5). The rest fungal homologues might have been lost in the early evolutionary years in the different fungal taxa, like in the case of Saccharomycotina, and regained through independent HGT events from bacterial homologues (Fig. 5). The reasoning for reacquiring this gene and in extent this protein could be the evolutionary advantage it offers in ecological niches, where AZC could provide for nitrogen or carbon source. Some of these niches are common between fungi and bacteria, since the majority of these fungal and bacterial species are soil residents. This association of habitat and gene gain or loss events, could be also a possible explanation for the absence of AzhA in Saccharomycotina, since these species are not residents of soil^74^.

Overall, in this work, we propose the existence of a number of AzhA ancestor homologue/s, as members of the HAD superfamily, which have been either transferred in several fungal, bacterial or archaeal species through HGT events, or, alternatively, the presence of an old homologue before the existence of the last common fungal ancestor, who might have been the mediator for the HGT events among the later species.

Finally, based on the presented data and in the absence of AzhA homologues in AZC-producing plants, we propose that AzhA hydrolase contributes to a defense mechanism developed by microorganisms coexisting with AZC-producing plants. Our phylogenetic analysis indicated the presence of AzhA in a) several human dermatophyta (such as *Histoplasma capsulatum, Blastomyces dermatidis* etc.), b) fungal phytopathogens of great agricultural importance (such as *Ustilago maydis* and *Magnaporthe oryzae* etc.) and c) in bacteria degrading toxic substances (*Candidatus* Colwellia aromaticivorans). In total, the identification of enzymes like AzhA and NgnA could provide the basis for the identification of new antifungal pharmaceutical compounds against important human and plant fungal pathogens. Additionally, AzhA could be used as a new positive selection marker for transformation in ΑΖC-sensitive yeast and plants^75,76^. Accordingly, as a member of the HAD family, AzhA could be tested for potential detoxifying properties upon other toxic compounds and could be used in synthetic biology, for the development of new detoxifying systems.

## Materials and methods

### Media and growth conditions

*A. nidulans* cells were cultured in appropriately supplemented minimal media (MM) or complete media (CM) as previously described^77,78^ (and Fungal Genetics Stock Center, http://www.fgsc.net/) and grown at 25 and/or 37 °C. More precisely, nitrogen sources, ammonium tartrate (NH^+^_4_), urea (U), proline (Pro), GABA and AZC were used at a final concentration of 5 mM. Carbon sources, D-glucose (Gl), D-fructose (Fr) and ethanol (EtOH) were used at a final concentration of 1%, 0.1% and 3% (w/v), respectively. β-alanine was used at a final concentration of 50 mM (see RNA isolation and RT-PCR section).

*S. cerevisiae* cells were grown at 30 °C on a minimal buffered medium, pH 6.1^79^, supplemented with galactose (Gal, 2% (w/v)) or glucose (Gl, 2% (w/v)) as carbon sources. Nitrogen sources, ammonium in the form of (NH_4_)_2_SO_4_, urea (U) or AZC were used at a final concentration of 5 mM. In *S. cerevisiae* the *A. nidulans azhA* and *ngnA* genes were under the control of the regulatable promoter *gal*1, which is repressed by glucose^80,81^.

## Strains and Plasmids

### *A. nidulans* strains and plasmids

The *A. nidulans* strains used in this study are listed in Table 1. Standard techniques for genetic analysis were used^82^. *A. nidulans* protoplast preparation and DNA transformation were performed as previously described^83^. For transformation of *A. nidulans,* DNA was isolated using the High Purity Plasmid kit (Roche), according to the manufacturer’s instructions and genomic DNA was prepared as described by Lee and Taylor (1990)^84^. The entire open reading frame of the *azhΑ* gene (ORF of AN12472) 726 bp long, was replaced in a recipient strain by the *Aspergillus fumigatus pyrG* gene (*Afpyrg)*, using the fusion PCR gene replacement method and standard molecular cloning techniques^85^. The recipient strain carries a deletion of the *nkuA* gene (*nkuA*Δ), which is responsible for non-homologous end joining of DNA fragments. Deletion of the *nkuA* gene decreases the frequency of non-homologous integration of transforming DNA sequences into the *A. nidulans* genome, leading to improved gene targeting up to 90%^83^. The ∼1500 bp long, 5′ and 3′ flanking sequences of the *azhA* gene were PCR amplified using the Kapa-HiFi (Kapa Biosystems) DNA polymerase and primers 1–2 and 3–4, respectively (Supplementary Table S1). PCR products were digested with *Not*I/*Xba*I and *Xba*I/*Kpn*I restriction endonucleases and subsequently cloned into a *Kpn*I/*Not*I linearized pBlueScript II SK(+) vector (Table 3). In the unique *Xba*I site of the resulting plasmid, the Af*pyrG* gene was inserted, following its PCR amplification using primers 5 and 6 (Supplementary Table S1) from plasmid p1439^85,86^ and *Xba*I digestion. The resulting cassette was PCR amplified using primers 1 and 4 and ∼2 μg of it, were used to transform the recipient strain. Selection of the transformants was carried out on MM with urea as sole nitrogen source. The intact, single copy, in-locus replacements were confirmed by Southern blot analysis using a non-radioactive, enzymatic DIG-labeled probe, prepared using the DIG Nonradioactive Nucleic Acid Labeling and Detection System **(**Roche), according to the manufacturer’s instructions. The *azhA*Δ *ngnΑ*Δ double deletion strain was constructed by *A. nidulans* standard genetic crossing techniques^87^.

**Table 1:** *A. nidulans* strains used in this study.

Strain	Genotype	References
*amdR*^*-*^	*biA1; pyroA4; niiA4; amdR-44*	Andrianopoulos and Hynes, 1990
*areA*^*600*^	*areA600; biA1; sB43*	Sealy-Lewis, 1987
*azhA*Δ	*azhAΔ::pyrG*^*Af*^*; nkuAΔ::argB; pyroA4; pyrG89; argB2*	This study
*azhA*Δ *ngnA*Δ	*azhAΔ::pyrG*^*Af*^*; ngnAΔ::pyrG*^*Af*^*; pyroA4 pantoB100; yA2*	This study
*creA*^*d*^	*creAd30; biA1*	Arst et al*.* 1990
*gabA*Δ	*gabAΔ::AfpyrG; pyrG89; pyroA4; nkuAΔ::argB*	Gournas et al*.*, 2015
*ngnA*Δ	*ngnAΔ::pyrG*^*Af*^*; nkuAΔ::argB; pyroA4 pyrG89 argB2*	Gournas et al*.*, 2015
*nkuA*Δ	*nkuAΔ::argB; pyrG89; pyroA4*	Laboratory collection^85^
***prnA-***		Laboratory collection
*prnB*Δ	*prn377; pabaA1; yA2*	Laboratory collection
*prnB*Δ *gabA*Δ	*prn377; gabAΔ::AfpyrG; pabaA1; pyroA4; yA2*	Gournas et al*.*, 2015^25^

### *S. cerevisiae* strains and plasmids

The *S. cerevisiae* strains used are listed in Table 2. They were constructed using the *Σ1278*b wild-type^88^ and BY4741 strains. Shuttle vectors, listed in Table 3, were used for the heterologous expression of *A. nidulans azhA* and *ngnΑ* genes into *Σ1278*b and BY4741 strains. Plasmids isolated were constructed by in vivo recombination in *S. cerevisiae* strains between linearized plasmids and PCR DNA fragments, using the primers reported in Supplementary Table S2^89^. Each plasmid was isolated from transformed *E. coli* cells and confirmed by sequencing.

**Table 2:** *S. cerevisiae* strains used in this study.

Strain	Genotype/description	References
*23344c*	*ura3*	Bruno André lab
*BY4741*	*ura3; his3; met25*	Bruno André lab
*S288C -ngn2*	*S* + *(pAA2)* + *(pRS416-GAL1) his3 met25*	This study
*S288C* + *pRF* + *pRS416*	*S* + *(pAA1)* + *(pRS416-GAL1) his3 met25*	This study
*S288C-azhA*	*S* + *(pAA1)* + *(pRS416-GAL1-azhA) his3 met25*	This study
*S288C-azhA-ngn2*	*S* + *(pAA2)* + *(pRS416-GAL1-azhA) his3 met25*	This study
*Σ-azhA*	*Σ1278b* + *( pRS416-azhA)*	This study
*Σ-pRS416*	*Σ1278b* + *( pRS416-GAL1)*	This study

**Table 3:** Plasmids used in this study.

Plasmid	Genotype/description	References
pBlueScript II SK(+)		Laboratory collection^85^
p1439	5GA-sgfp-AfpyrG	Laboratory collection^85^
pAA1	pFL36-GAL1	This study
pAA2	pFL36-GAL1-ngnA	This study
pRS416-GAL1	pRS416-GAL1	Bruno André lab
pRS416-azhA	pRS416-GAL1-azhA	This study

### Microscopic observation of growth rate

Time lapse confocal microscopy was used for growth rate observations. Spores from freshly inoculated CM were collected and each spore suspension was used to inoculate supplemented liquid MM in µ-Slide 8 Well Chamber Slide (Ibidi GmbH, Germany). Cells were incubated for 18 h at 25 °C and observed by the use of a Leica TCS SP8 (Leica Microsystems Ltd., Milton Keynes, UK) confocal microscope. Growing hyphal tips were recorded with brightfield microscopy at high magnification, using a Leica HC PLΑΝ APO 63x/1.40NA oil immersion objective, and digital images captured every 15 min for a total of 45 min. Following, registration was performed automatically using the ImageJ plug-in, Linear Stack Alignment with SIFT, and tip growth rates were calculated using the MTrackJ manual tracking plug-in^90^ in Fiji image processing package^91^. At least 40 cells were counted for every condition tested at each experiment. More precisely, the total number of cells (n) observed for each condition tested was: WT: Urea, n = 161; Urea + AZC, n = 168; AZC, n = 171, *azhA*Δ: Urea, n = 120; Urea + AZC, n = 178; AZC, n = 126, *ngn2Δ*: Urea, n = 301; Urea + AZC, n = 202; AZC, n = 273, *azhAΔ ngn2*Δ: Urea, n = 271; Urea + AZC, n = 318; AZC, n = 362).

### RNA isolation and RT-PCR

For total RNA extraction, *A. nidulans* conidiospores from ¼ of a 4-day CM plate were suspended in MM, filtered through blutex, and used to inoculate appropriately supplemented liquid MM. Mycelia were grown with shaking and subsequently harvested as previously described^92^. Where indicated, carbon and nitrogen sources were added as follows: D-glucose at a final concentration of 1%, D-fructose at 0.1%, EtOH at 3%, urea at 5 mM and ammonium, as tartrate salt at 5 mM. For induction, L-Pro, GABA or AZC were added at a final concentration of 20 mM and β-alanine at 50 mM. Mycelia were incubated at 25 or 37 °C for a total of 16 h. In order to examine the *gatA* gene expression, mycelia were grown at 37 °C for 26 h on MM with fructose and ammonium tartrate as carbon and nitrogen sources, respectively, as previously described by Richardson et al. (1989)^27^. Following, mycelia were thoroughly washed with ice-cold MM and about 1 g (net weight) was transferred aseptically in MM with fructose as carbon source, and 50 mM β-alanine or 20 mM AZC as nitrogen sources, respectively and incubated for additional 4 h.

RNA samples were prepared as previously descripted^92,93^, using the TRI Reagent (Sigma-Aldrich) kit, according to manufacturer’s instructions. Moreover, to avoid contamination with genomic DNA, 10 μg of each RNA sample were treated and cleaned up with TURBO DNA-free kit (Invitrogen). The absence of DNA contamination was verified by a conventional PCR (approximately 30 cycles), using specific- *azhA, ngnA, gatA* and 18S rRNA primers (Supplementary Table S1, primers 7 to 14,) and at least 100 ng of each RNA sample as template. The quality of isolated RNAs was examined by conventional gel electrophoresis using a 2% agarose gel stained with ethidium bromide (0,5 μg/ml). The concentration of each RNA sample was calculated using Nanodrop equipment (ND-1000 Spectrophotometer) according to the instructions of the manufacturer.

Approximately 100 ng, of each RNA sample, were used for reverse transcription PCR (RT-PCR) using the PrimeScript RT master mix (Perfect Real Time) (Takara Bio), according to manufacturer’s instructions. Primers for RT-PCR were designed so that the amplification fragment includes sequences belonging to at least one intron of each gene tested (except for *azhA* which is an intron-less gene) and were expected to produce amplicons of 355 bp (*azhA* cDNA), 478 bp (*ngnA* cDNA), 371 bp (*gatA* cDNA) and 280 bp (18S ribosomal RNA gene) (Supplementary Table S1). Densitometry analysis of RT products on an EtBr-stained 1% agarose gel was performed using ImageJ 1.43 (NIH) software. Relative optical density was calculated by dividing the densitometry of gene-specific cDNA with the respective loading control (18S rRNA).

### Statistical analysis

Data were presented as mean ± SEM. GraphPad Prism software, version 8.0 was used for all statistical analyses. Two-way ANOVA with the nonparametric Kruskal–Wallis test, and Dunn’s multiple-comparison post hoc analyses were used to assess the significance of the value differences of all measurements.

### Data collection

The Basic Local Alignment Search Tool for proteins (BLASTp) was used to retrieve amino acid sequences similar to AzhA, across the three main phylogenetic Domains (Archaea, Bacteria and Eukaryotes). AzhΑ hydrolase amino acid sequence was used as the query sequence throughout the NCBI database (retrieved on September 2019). The parameters used for sequence selection were: E-value threshold 10^–05^ maximum, query coverage 75% minimum and percentage identity at least 25%, using the default algorithmic settings to run the blast. In classes, where, too many sequences were retrieved, sequences of maximum homology according to the mentioned criteria were selected by all the representative orders.

### Multiple sequence alignment

The collected sequences were aligned using the Clustal Omega (https://www.ebi.ac.uk/Tools/msa/clustalo)^94^ and the Jalview Version 2 programs^95^. Alignment parameters were set to default and results were inspected and manually edited in the sequence editor BioEdit, when necessary (http://www.mbio. ncsu.edu/BioEdit/bioedit.html, last accessed September 2019).

### Phylogenetic analysis

Molecular evolutionary analyses were conducted by Neighbor Joining analysis (NJ), using the PAUP program^96^ and by Bayesian analysis, using the MrBayes program^95–99^. Reliability of nodes was assessed using 10 M bootstrap iterations for the NJ analyses. For the Bayesian analysis, the model of protein evolution that best fits the AzhA amino acid multiple sequence alignment was selected using the ProtTest program^100^. For the matrix containing all the fungal sequences the best fitting model (LG + I + G + F; alpha = 1) was determined according to BIC (Bayesian Information Criterion) and DT (decision theory). For the matrix containing AzhA similar sequences from all organisms, the best fitting model (LG + I + G + F; alpha = 1.47) was determined according to AIC (Akaike Information Criterion). The ProtTest program was also used for the estimation of the proportion of invariable sites and the alpha parameter of the gamma-distributed substitution rates. In all matrices (i.e. the matrices from all fungal strains and from all organisms), four independent MCMC searches were performed for each data set employing different random starting points (number of generations = 5,000,000), with sampling every 5000 generations. Convergence was inspected visually by plotting likelihood scores versus generation for the two runs. Based on this analysis, the burn-in was set to 10,000 in all cases. The program FigTree was used for the visualization of the trees and to make changes for their better presentation^101^ and phylo.io^102^ was used for the comparison of the trees constructed by the two different methods (data upon request). In all produced cases an automated comparison was conducted with plylo.io and the trees were found similar and most importantly identical at the topologies analyzed in the Results section.

## Supplementary Information

Supplementary Information 1.

Supplementary Video 1.

Supplementary Information 2.
