# Supplementary material for: A highly conserved mechanism for the detoxification and assimilation of the toxic phytoproduct L-azetidine-2-carboxylic acid in Aspergillus nidulans
Source: Sci Rep. 2021 Apr 1;11:7391. doi: 10.1038/s41598-021-86622-3 (PMC8016842; doi:10.1038/s41598-021-86622-3)
Supplement: Supplementary file 1 — Supplementary Information 1. [file 41598_2021_86622_MOESM1_ESM.pdf]

Supplementary Information

A highly conserved mechanism for the detoxification and assimilation of the toxic phytoproduct L-azetidine-2-carboxylic acid in *Aspergillus nidulans*.

Ada Biratsi,[a] Alexandros Athanasopoulos,[a,c] Vassili N. Kouvelis,[b] Christos Gournas,[a] and Vicky Sophianopoulou[a]#

[a]Microbial Molecular Genetics Laboratory, Institute of Biosciences and Applications, National Centre for Scientific Research, Demokritos (NCSRD), Athens, Greece.

[b]Department of Genetics and Biotechnology, Faculty of Biology, National and Kapodistrian University of Athens, Athens, Greece

[c] Optical Microscopy Unit, Institute of Biosciences and Applications, National Centre for Scientific Research, Demokritos (NCSRD), Athens, Greece.

#Address correspondence to vicky@bio.demokritos.gr

Supplementary Table S1: Oligonucleotides used in this study for *A. nidulans*

Name	Sequence (5'to 3')
1. 5'AN12472NotIFor	attcgcggccgcCAGACAAGCGGCTCATAG
2. 5'AN12472XbaIRe	atcttctagaGTGTTGTTGACAGGAGAGC
3. 3'AN12472XbaIFor	cttatctagaGTATGGCGAGGGTGAATG
4. 3'AN12472Rev	GGCATGGTGAATTGGGTTC
5. AfpyrG XbaI F	CGCGTCTAGAGCCTCAAACAATGCTCTTCACCCTC
6. AfpyrG XbaI R	CGCGTCTAGACTGTCTGAGAGGAGGCACTGATGCG
7. azhART Fr	GCTCATATCGCCTCCTAACC
8. azhART Rv	GGCCTCACAATCCAGACACT
9. ngnA_RT_Fr	GCCCATCATCTCTTCCACAG
10. ngnA_RT_Rv	GTACGCCTTGCCCATCAC

11. 18SrRNA_RT_Fr	CCGTTCTTAGTTGGTGGAGTG
12. 18SrRNA_RT_Rv	GCTCTATCCCCAGCACGACA
13. gatA_RT_Fr	CTTCCCCAATGAGCCTACTG
14. gatA_RT_ Rv	GGTAAACACCTGGTCCAAGC

Supplementary Table S2: Oligonucleotides used in this study for *S. cerevisiae*

**Name**	**Sequence (5’to 3’)**
1. S.c.-azhA-Fr	TGTTAATATACCTCTATACTTTAACGTCAAGGAGAAAAA ACTATAATGTCATTAAGCTCATATCGCC
2. S.c.-azhA-Rv	TGACATAACTAATTACATGATATCGACAAAGGAAAAGG GGCCTGTCTATAAAGTTACAGCTGCCTC
3. S.c.-ngnA-Fr	TGTTAATATACCTCTATACTTTAACGTCAAGGAGAAAAA ACTATAATGCCCTCTATCCTTGAAGA
4. S.c.-ngnA-Rv	TGACATAACTAATTACATGATATCGACAAAGGAAAAGG GGCCTGTCTAGGCGTTTTCTACTACAG
5. Fr.His.S.c	CACGACGCTTTGTCTTCATTC
6. Rv,His.S.c	CTCGTTGTTGTCGTTGATGC
7. Fr.Meth.Sc	CGATATTGGGGAACTGGGTG
8. Rv.Meth.S.c	CCCCGAAGTTTTCCTGATT

Supplementary table S3. BLAST outputs using different AzhA homolog against different databases
(see Excel file: Supplementary_Table_S3)

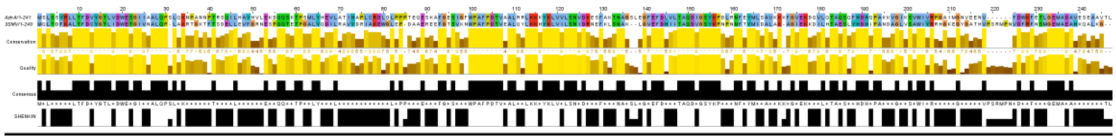

Supplementary Figure. S1. Sequence alignment of the AzhA and AC2 *Pseudomonas* hydrolase.

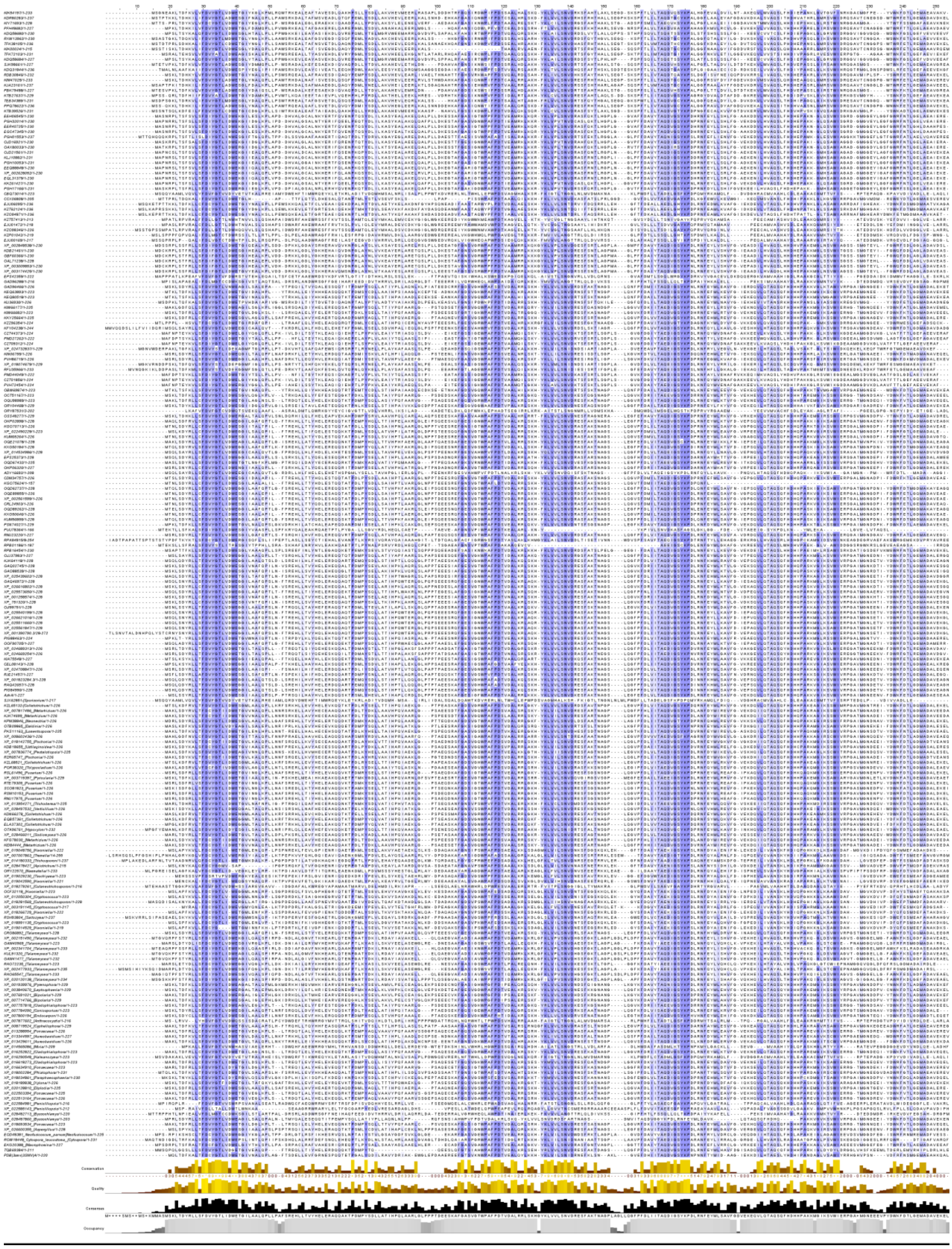

Supplementary Figure. S2. Multiple sequence alignment of AzhA homologs among representative taxa of Fungi indicating the conservation of specific residues and motifs.

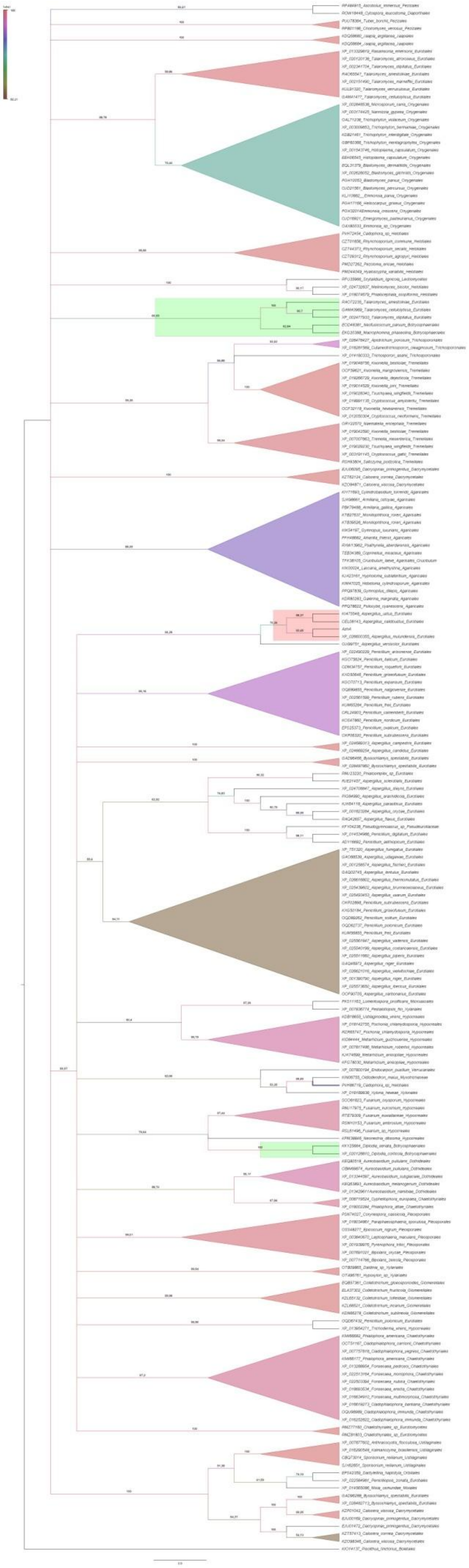

Supplementary Figure. S3. Phylogenetic relationships of AzhA homologs among representative taxa of Fungi using the NJ analysis method. NJ- Bootsrap support is represented, as follows; red: values from 100% to 99% (in all cases), purple: values ranging from 98% to 89%, blue: values ranging from 88% to 73%, green: values ranging from 72% to 57% and brown: values ranging from 56% to 51%.

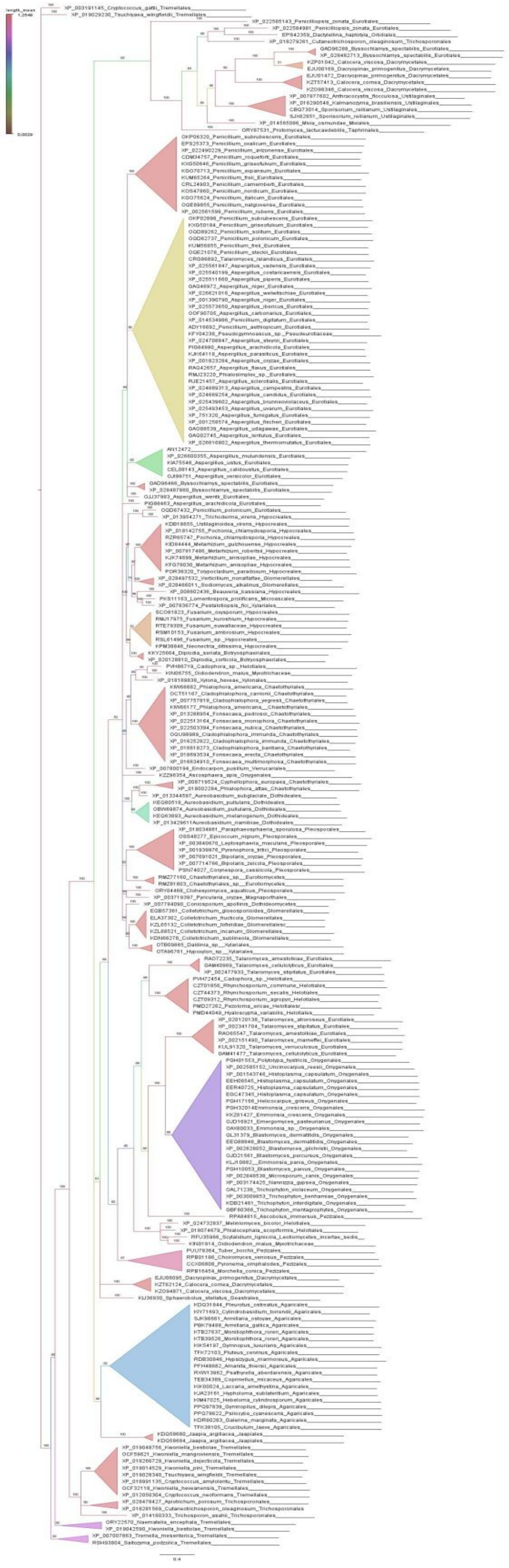

Supplementary Figure. S4. Phylogenetic analysis of AzhA homologs among representative taxa of Fungi using Bayesian analysis method.

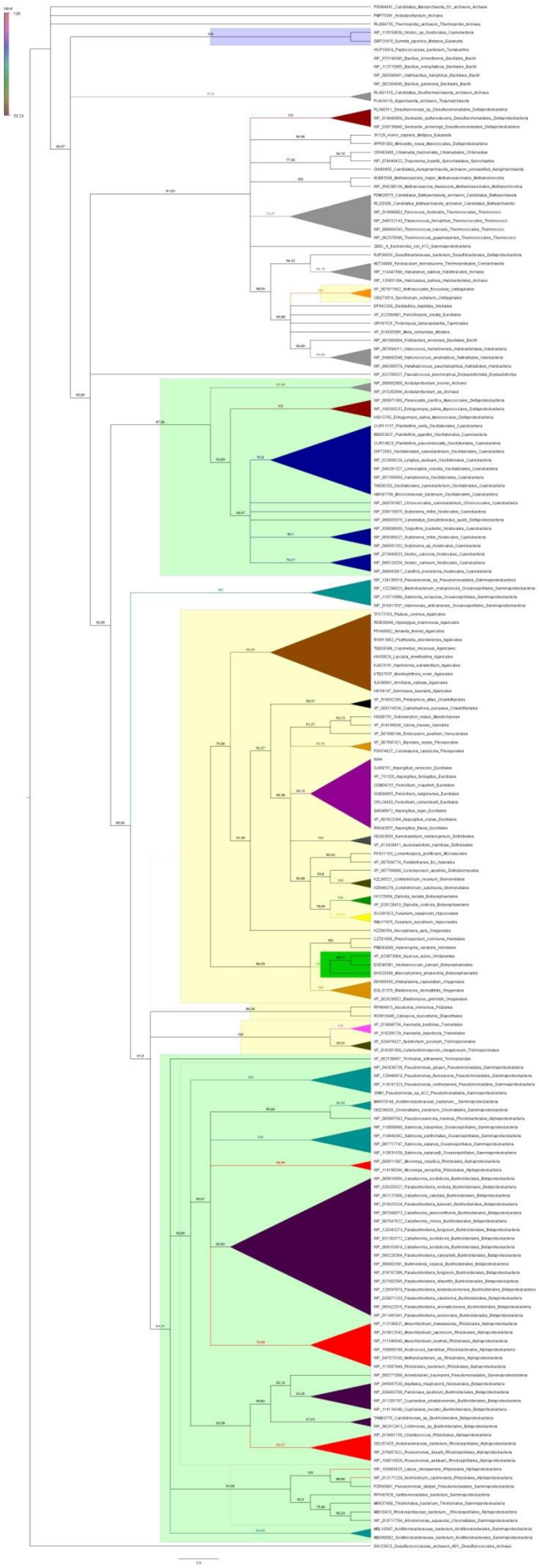

Supplementary Figure S5. Phylogenetic relationships of AzhA homologs among representative taxa of Fungi, Bacteria and Archaea, using the NJ analysis method. NJ-Bootstrap support is depicted. The *Aciduliprofundum* spp. were used to root the tree. The tree is divided into two big clusters I and II (highlighted with pink and blue, respectively).

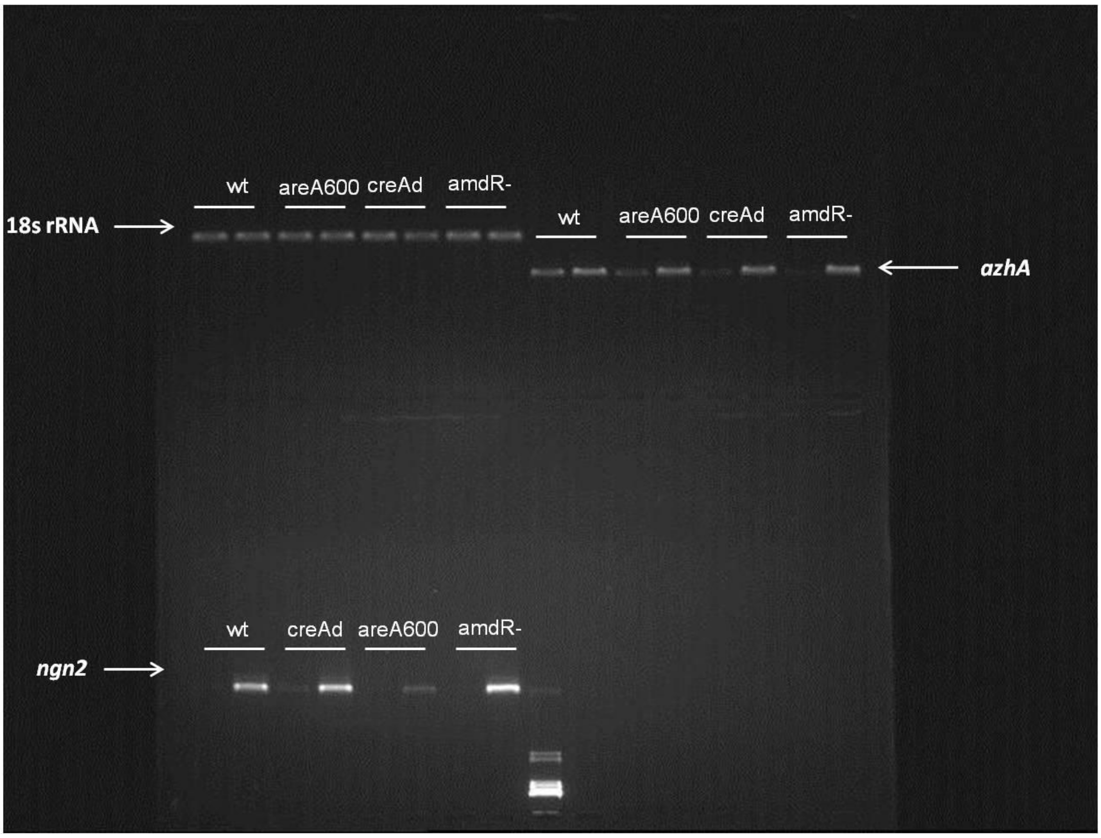

Supplementary Figure S6. Full length agarose gel of Figure 2A.

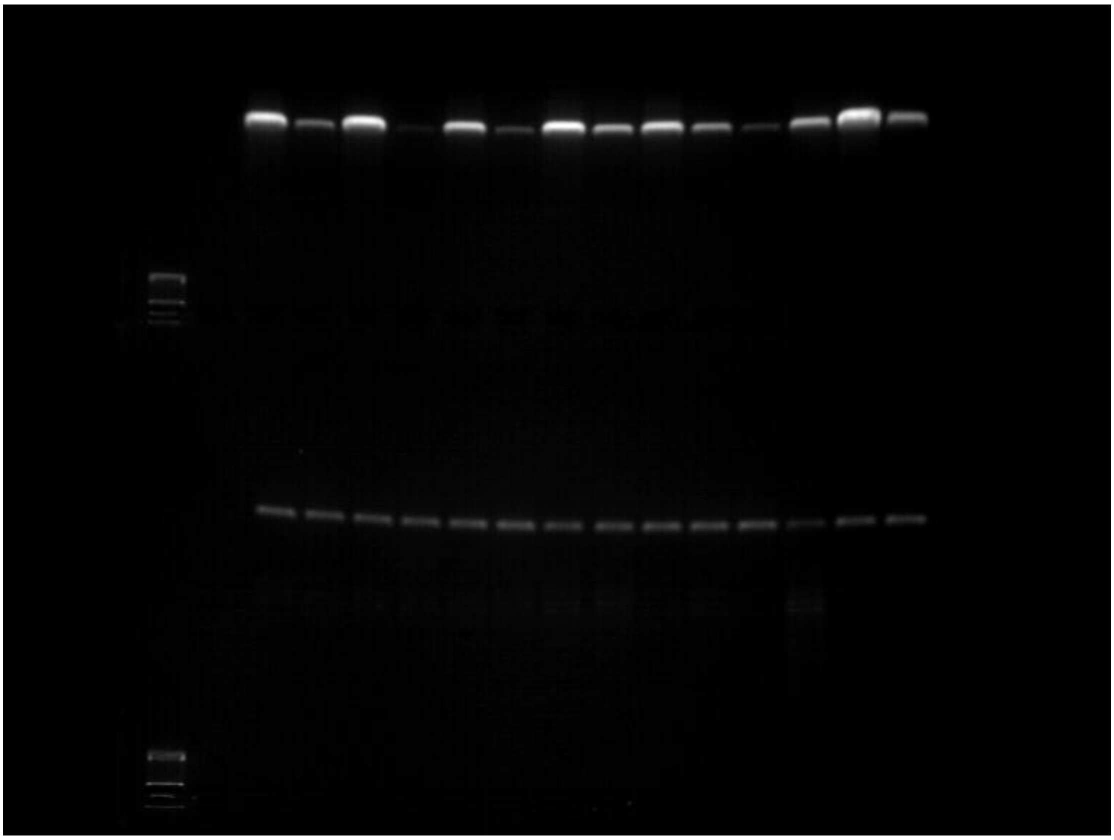

Supplementary Figure S7. Full length agarose gel of Figure 2B.

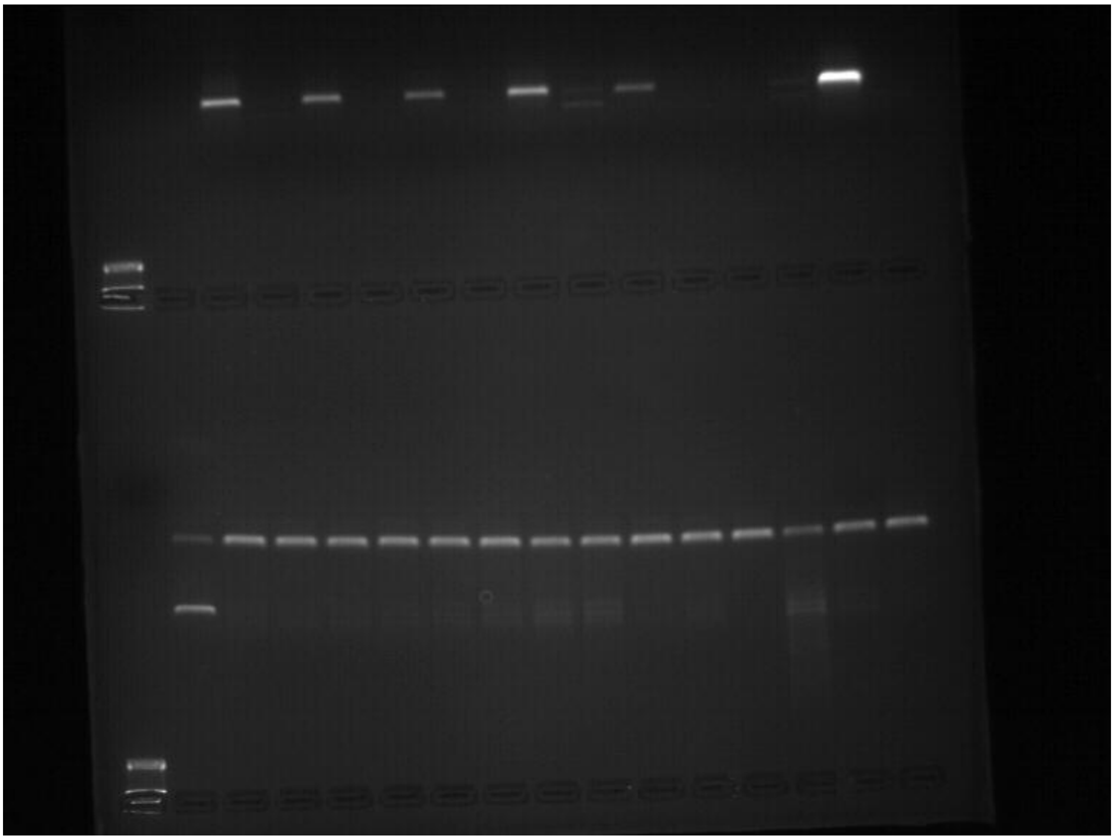

Supplementary Figure S8. Full length agarose gel of Figure 2C.

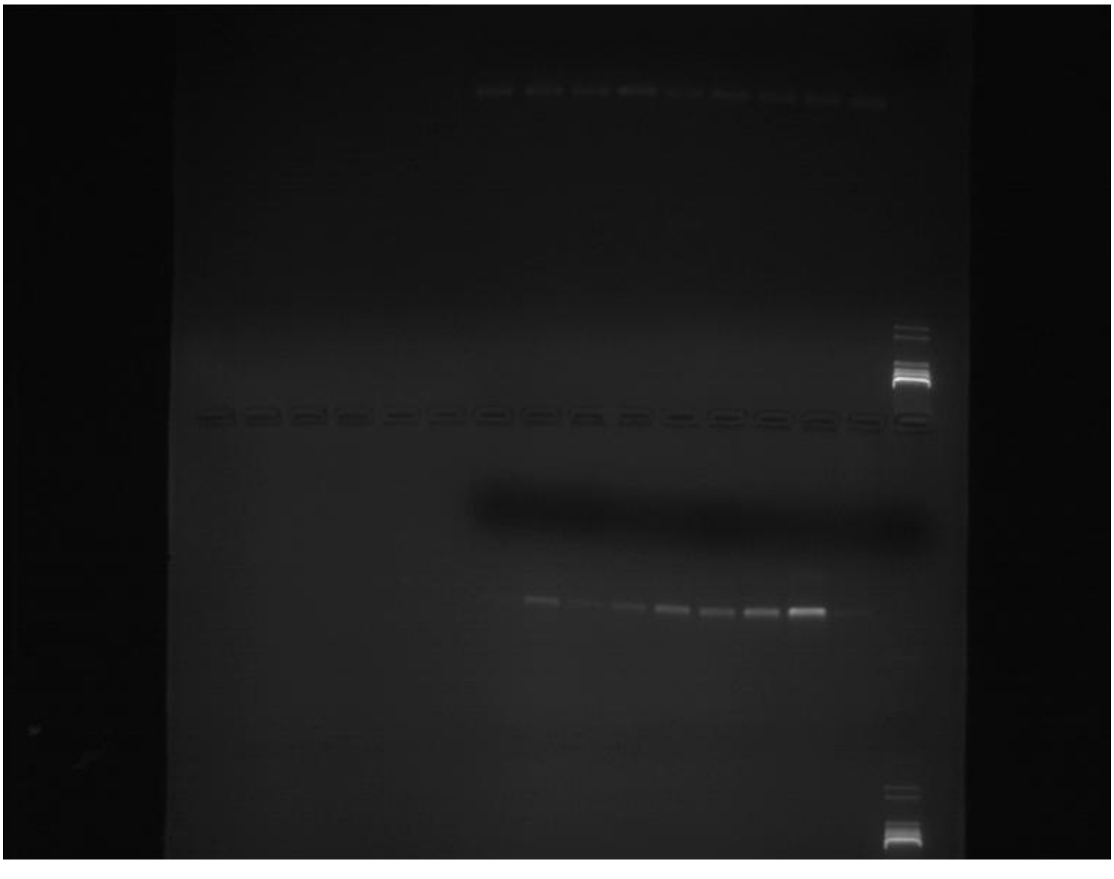

Supplementary Figure S9. Full length agarose gel of Figure 3A.

Supplementary Video. S1. Growth of *A. nidulans* strains observed in liquid cultures, containing different nitrogen sources. Growth rate differentiation of WT, *azhA*Δ, *ngn2*Δ single mutant and *azhA*Δ *ngn2*Δ double mutant strains is presented, on different nitrogen sources, at different time frames (every 15 minutes for a total of 45 minutes). Liquid MM were supplemented with urea (U) and/or L-AZC at a final concentration of 5 mM each. All strains were incubated for a total of 18 h, at 25 ^{0}C.
